# Bulk tank milk ELISA as screening test for *Mycoplasma bovis*: herd classification based on serology and PCR testing of different age groups

**DOI:** 10.1186/s13620-025-00327-x

**Published:** 2026-01-17

**Authors:** Jolien Vandewalle, Wouter van Mol, Jade Bokma, Charlotte Rigauts, Bart Pardon, Stan Jourquin

**Affiliations:** 1 Calf Health Research Group, Department of Internal Medicine, Reproduction and Population Medicine, Faculty of Veterinary Medicine, Ghent University, Salisburylaan 133, Merelbeke, 9820 Belgium; 2 Animal Health Services Flanders (DGZ Vlaanderen), Industrielaan 29, Torhout, 8820 Belgium; 3 Veterinary Practice Venhei, Geelsebaan 95, Kasterlee, 2460 Belgium

**Keywords:** *Mycoplasma bovis*, Herd status, ID-Screen, Screening strategy

## Abstract

Herd-level infection status for *Mycoplasma bovis* is commonly assessed by detecting antibodies in bulk tank milk (BTM) samples. However, the association between BTM serology and active circulation of the pathogen at farm level remains largely unclear, in part because non-lactating animals, and particularly calves, fall outside this sampling strategy. This cross-sectional pilot study aimed to categorize 14 dairy herds based on the ID-screen BTM antibody ELISA for *M. bovis*, and compare those results with PCR testing and serology in adult cows, and PCR testing in youngstock and calves. Serum sample-to-positive percentage (S/P%) from adult cows was measured using the Bio K432 ELISA. Nasal swabs (NS) from adult cows, calves, and youngstock, and vaginal swabs (VS) from lactating cows, were pooled by age group and tested by PCR. Based on manufacturer’s guidelines and optimized cutoff values for the ID-screen ELISA, herds were categorized as BTM negative (S/P% <30%; *n* = 5), BTM positive (S/P% ≥50%; *n* = 6) or BTM indeterminate (S/P% 30–50%; *n* = 3). Median serum S/P% was 26.3 (Interquartile range (IQR); 15.9–42.4), 81.33 (IQR; 52.7-108.8) and 43.4 (IQR; 27.5–95.0) in BTM negative, positive and indeterminate herds, respectively. PCR detected *M. bovis* only in pooled NS samples from calves and youngstock in two BTM positive herds. Seroprevalence aligned with BTM results for positive and negative herds, but varied in indeterminate herds. BTM testing may serve as a valuable screening tool to distinguish herds likely free of *M. bovis* from those where additional testing of calves and youngstock is recommended.

**Supplementary Information:**

The online version contains supplementary material available at 10.1186/s13620-025-00327-x.

## Introduction

*Mycoplasma bovis* (more recently named *Mycoplasmopsis bovis;* [1]) causes disease in cattle of all ages, and is commonly associated with pneumonia, arthritis, otitis and mastitis [2]. The infection often becomes chronic, leading to high antimicrobial use, compromised animal welfare and important economic losses in all cattle industries worldwide [3, 4]. For control, certification and eradication programs, establishing a herd-level infection status is highly beneficial, as it can assist in purchase decisions and guide preventive measures. A practical method to screen dairy herds is to monitor bulk tank milk (BTM) for antibodies (e.g. ELISA) or genetic material (e.g. PCR, culture) [5, 6]. Antibody ELISA on BTM is often preferred over PCR in national programs, as it is more cost-efficient, does not require active shedding at the time of sampling and the longevity of antibody expression can last for 4–18 months [6–10]. In literature, different ELISA-kits have been tested on BTM, such as: ID-screen (e.g. ID-Vet, Grabels, France), Bio K260, Bio K302 and Bio K432 ELISA kits (Bio-X Diagnostics, Rochefort, Belgium), and an in-house MilA ELISA [11–13]. In a recent study comparing the ID-screen, Bio K432 and Bio K302 kits using Bayesian latent class modeling, the ID-screen was shown to have the highest sensitivity (Se) when using the manufacturer’s cutoff (estimated Se and specificity (Sp) of 91.4% and 67.2%, respectively), and having both the highest Se and Sp when using optimized cutoffs (estimated Se and Sp of 89% and 83.4%, respectively) [13]. However, despite the apparent potential of using the ID-screen ELISA for screening purposes (high Se), and even its use in national programs (e.g. New Zealand), there is currently no information available about the association between BTM and the presence of antibodies and antigen in different age groups. Because calves, often the main drivers of *M. bovis* transmission due to their higher susceptibility, subclinical nature of disease and close-contact spread, are not included in BTM testing, the suitability of this method for whole-herd screening is questionable [14, 15]. Also, it is often unclear how test results surrounding the cutoff values should be interpreted. When using BTM, minimizing false negatives is crucial to prevent introduction of disease into naïve herds and prevent spread within the herd caused by undetected carriers. In contrast, false positive results can cause unnecessary concerns and economical losses (e.g. unnecessary additional testing and culling). Although optimizing ELISA cutoffs has been described as a method to increase Sp (minimizing false positives), there is currently no established approach to assess whether borderline BTM results truly reflect the presence of antibodies and active circulation in the herd and whether additional testing in these herds may be warranted. Therefore, the objective of the current study was to explore the potential of the ID-screen BTM antibody ELISA as a screening test for *M. bovis* in dairy herds, using the manufacturers and optimized cutoff values, by comparing BTM ELISA test results with serology and PCR testing in individual cattle (lactating and dry cows), and PCR testing in youngstock and calves.

## Material and methods

### Study population and sampling strategy

A cross-sectional study was conducted between January and December 2024 in Belgium. In collaboration with the Flemish Animal Health services (DGZ Vlaanderen), a call was launched via a newsletter for farmers interested in determining the *M. bovis* status of their herds. To participate, farms had to be dairy operations with a minimum of 40 animals and had to be involved in the national dairy herd improvement program (Melkcontrole, MCC, Vlaanderen). For this study, only the first 50 farms that applied and met the abovementioned criteria were included. A BTM sample was collected during routine milk collection from each of these farms and analyzed using the ID-Screen ELISA test (ID-Vet, Grabels, France). From these 50 herds, a selection of 14 farms was made based on herd size (≤ 150 cows in lactation), willingness to further participate and BTM Sample-to-positive percentage (S/P%) (e.g. strongly positive, strongly negative or within a borderline range). None of the selected herds had reported clinical disease compatible with *M. bovis* (e.g. pneumonia outbreaks or clinical mastitis attributed to *M. bovis*) in the months prior to sampling. This absence of reported disease was confirmed through farmers interviews, herd health records and clinical presentation of the animals at the time of sampling. In addition, a second BTM sample was taken and analyzed within one week before or after the farm was visited, and the BTM results closest to the sampling were used for analysis. During the visit, samples were taken from the following groups of animals: adult cows (> 24 months, further split into lactating and dry cows), youngstock (6–24 months) and calves (< 6 months). Based on the number of animals present in each group, the sample size was calculated following the ‘detection of disease’ principle using WinEpiScope (WinEpiScope, version 2.0). The confidence interval was set at 95% and the number of infected animals to detect was set at five. An exception was made for two herds; for one herd (herd 14), sample size was reduced to 40 instead of the calculated sample size of 65 for practical reasons. In another herd (herd 7), no dry animals were present at the moment of sampling. All animals were randomly selected from the national register, with the list organized by age and production type. Each animal was assigned a random number using Excel rand function, and those with the highest numbers were chosen until the desired sample size was reached for each age group. Following the approach described by [16], assuming calvings are evenly distributed throughout the year and dry periods account for roughly 10% of the year, the number of dry cows was estimated as 10% of the adult cow population. However, in seven herds, the number of dry cows sampled was reduced to 10% of the sample size of the lactating cows. This reduction was necessary due to budgetary constraints and the fact that these herds had a more seasonal calving pattern, resulting in a limited number of dry cows available for sampling. Herds were sampled between March and December 2024. The study protocol was approved by the Ethical Committee of the Faculty of Veterinary Medicine and Bioscience Engineering of Ghent University under license number EC 2023-070. The study was conducted and reported following STROBE guidelines.

### BTM analysis

A BTM sample was collected during routine milk collection of the 50 herds that were included and analyzed using the ID-Screen ELISA test (ID-Vet, Grabels, France), as this was the standard ELISA for BTM testing used by the national animal health service laboratory, and thus relevant to verify for application in the region the study was performed. For the ID-screen the overnight protocol was used. Analysis was performed following manufacturer’s instructions, with the protein G peroxidase as the conjugate. The S/P% for each sample and test was calculated using the following formula:

$$\:S/P\%\:=\:\left(\frac{ODsample\:-\:ODmean\:negative\:control}{ODmean\:positive\:control\:-\:ODmean\:negative\:control}\right)x\:100$$

A combination of both manufacturer (S/P% <30%) and optimized cutoff values (S/P% ≥50%) as defined by Bokma et al., (2024) was used to determine whether a BTM sample was strongly positive, strongly negative or fell into a borderline range. Based on these classifications, 14 herds were selected to ensure representation of all three BTM categories. The BTM results for the 14 selected herds were as followed: five BTM samples were labeled strongly positive (S/P% values > 70%), five BTM samples were labeled strongly negative (S/P% values < 25%) and four BTM samples had S/P% values just below or slightly above the 50% cutoff (three BTM samples < 50% and one BTM sample > 50%), falling into the borderline range.

### Serology

Blood samples were taken from the coccygeal vein of adult cows using a 21G needle and vacutainer (Venoject, Terumo, Leuven, Belgium). Samples were kept in a container (temperature of 4–7 °C) and arrived at the laboratory within 24 h after collection. All serum samples were analyzed with the Bio K432 ELISA kit (Bio-X Diagnostics, Rochefort, Belgium), as this was the standard ELISA used by the national animal health service laboratory, following manufacturer’s instructions. The conjugate used in Bio K432 was the monoclonal α-IgG2 peroxidase. The S/P% for each sample and test was calculated using the same formula described above.

### PCR testing on nasal and vaginal swabs

Both nasal (all tested animals) and vaginal swabs (VS) (lactating animals) were collected using a 15 cm sterile, dry swab (Deltalab, Barcelona, Spain). In calves and youngstock, a 15 cm swab reaches the nasopharynx and is referred to as a deep nasopharyngeal swab (DNS), whereas for adult cows, the same 15 cm swab was classified as a nasal swab (NS). Before inserting the swab into the nose, the nose was rinsed with isopropylalcohol applied on cotton wool. The swab was inserted approximately 15 cm deep into the right nostril, rotated and then immediately inserted into the left nostril. Both for the DNS and NS the same swab was used to sample both the right and left nostril in each animal, as this has been described to be an effective strategy to increase the likelihood to retrieve a positive sample [17]. Immediately after sampling, a maximum of five swabs, depending on the sample size per age group, were stored together in the same sterile tube, containing 5mL of sterile 0.9% NaCl. Each of these tubes was defined as a different pool sample. Of the cows in lactation, a VS was collected. Before inserting the swab into the vagina, the vulva was cleaned with a 0.05% chlorhexidine solution (Coditane 5%^®^, Kela, Hoogstraten, Belgium). Pooling of the VS was performed using the same procedure as that applied for the (D)NS. No VS were taken from the dry cows at the farmer’s request due to concerns about possible effects on the impending calving. All samples were stored in a container (temperature of 4–7 °C) and transported to the laboratory within 24 h after collection. Each pool of both (D)NS and VS was analyzed for the presence of *M. bovis* using a commercially available real-time PCR (BactoReal^®^ Kit *Mycoplasma bovis*, Ingenetix, Vienna, Austria), following the manufacturer’s recommendations. Cycle thresholds (Ct) were determined for each sample. A Ct-value between 37 and 45 was used to define an indiscriminate result, while a Ct-value < 37 was interpreted as positive. If no Ct-values were obtained, the sample was considered as negative.

### Data analysis

All animal data and test results were entered into Excel (Microsoft Inc., Redmond, Washington, USA) and transferred to SPSS statistics Version 29.0 (IBM Corp., Armonk, NY, USA) for analysis. First, farms were categorized based on BTM ELISA results, using a combination of the manufacturer’s guidelines and the optimized cutoff values described by [13], into one of three categories: BTM negative (S/P% <30%; *n* = 5), BTM positive (S/P% ≥50%; *n* = 6) and BTM indeterminate (S/P% 30–50%; *n* = 3). Secondly, serostatus was determined using the manufacturer’s cutoff for the Bio K432 ELISA (positive if S/P% ≥80 (Bio K432_≥ 80_). In addition, samples were also classified using an optimized cutoff (positive if S/P% ≥50 (Bio K432_≥ 50_) [18]. For each herd, the prevalence of seropositive animals was calculated for each category using both cutoff values. Next, the number of positive pooled PCR samples was determined for each farm. For each BTM category, the distribution of the serum S/P% was evaluated for a normal distribution by inspection of the histogram, and tests of normality were performed using the Kolmogorov-Smirnov test. Significance was set at *p* < 0.05. If no normal distribution was found, the data was analyzed using the median (Med) and Interquartile range (IQR) instead of the mean and standard deviation (SD).

## Results

Of the initial 50 herds, 14 herds, with a total of 707 animals, were included in this pilot study. An overview of BTM ID-Screen S/P% values for the initial 50 herds is provided in Supplementary Table S1. The 14 included herds had a mean herd size of 126.6 animals (Standard Deviation (SD) = 43.8; Minimum (Min) = 77; Maximum (Max) = 232). On average, herds comprised 77.4 adults (SD = 29.8; Min = 46; Max = 144), 34.7 youngstock (SD = 12.4; Min = 18; Max = 60), and 14.5 calves (SD = 6.2; Min = 7; Max = 28). An overview of the number of sampled animals from each age group per herd is provided in Table 1. 

**Table 1:** Overview of the number of animals that were sampled in 14 Belgian dairy herds with different levels of BTM antibodies against *Mycoplasma bovis*, stratified by production category

Herd	BTM S/P% ID-Screen	N° adult cows sampled (sampled/total present)	N° youngstock sampled (sampled/total present)	N° calves sampled (sampled/total present)	N° DNS	N° NS	N° VS	N° blood samples
BTM negative (S/P% <30%)								
3	4.2	29/66	20/46	7/17	27	29	24	29
1	4.5	21/47	6/14	6/15	12	21	15	21
4	4.97	30/68	11/26	7/16	18	30	29	30
2	12.53	22/50	14/33	4/10	18	22	17	22
6	24.01	25/57	11/26	5/13	16	25	20	25
BTM indeterminate (S/P% 30-50%)								
7	46.1	16*/48	8/19	2/6	10	16	16	16
5	48.97	58/130	16/36	7/17	23	58	45	58
13	48.98	29/65	17/39	6/14	23	29	26	29
BTM positive (S/P% ≥50%)								
10	54.59	24/54	11/26	3/8	14	24	21	24
8	70.26	37/82	13/30	3/7	16	37	33	37
12	71.74	42/95	18/41	6/15	24	42	38	42
14	87.99	40**/144	27/60	11/26	38	40	36	40
11	89.18	29/66	15/35	6/15	21	29	26	29
9	89.29	33/75	9/21	3/7	12	33	30	33

*Note*: *only lactating animals were sampled because no dry animals were present at the moment of sampling, **sample size was reduced to 40 instead of the calculated sample size of 65 for practical reason

Of these 14 herds, a second BTM sample was taken within one week of the visit to verify their BTM category. For herd 13, BTM S/P% had decreased markedly, resulting in reclassification into the BTM indeterminate group instead of the BTM positive group. All other herds remained within the same category. The median serum S/P% of animals in BTM negative, BTM indeterminate and BTM positive herds was 26.3 (IQR = 15.9–42.4; Min = 1.2; Max = 262.2), 43.4 (IQR = 27.5–95; Min = 7.0; Max = 226.8 ) and 81.3 (IQR = 52.7-108.8; Min = 12.5; Max = 188.3), respectively. An overview of the serum S/P% in the different BTM categories for all tested animal groups is provided in Table 2. 

**Table 2:** Overview of serum S/P% in 14 Belgian dairy herds with different levels of BTM antibodies against *Mycoplasma bovis*, stratified by production category

BTM – category*	Number of herds in category	Serum adults** S/*P*%*** (Median [IQR])	Serum lactating S/*P*% (Median [IQR])	Serum dry S/*P*% (Median [IQR])
Negative (S/P% <30%)	5	26.3 [15.9–42.4]	27.5 [16.8–45.4]	19.9 [12-37.1]
Indeterminate (S/P% 30–50%)	3	43.4 [27.5–95]	41.1 [26–95]	59.6 [40.9-108.8]
Positive (S/P% ≥50%)	6	81.3 [52.7-108.8]	80.9 [52.3-107.9]	87.6 [53.9-119.2]

*Abbreviations*: *S/P%* Sample-to-positive percentage, *BTM* Bulk tank milk, *IQR* Interquartile range

*by using the ID-Screen ELISA

**adults = sum of lactating and dry animals

***by using Bio K432

**Fig. 1:**
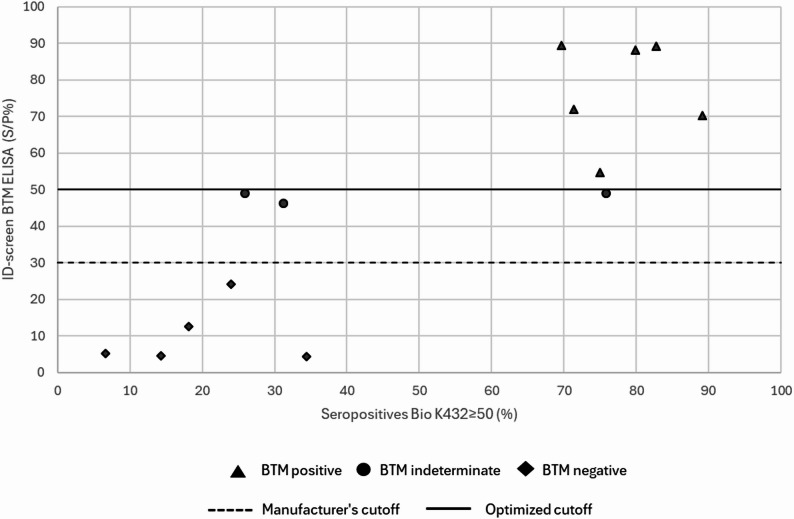
The distribution of the 14 herds, classified by ID-screen ELISA results on bulk tank milk (BTM) using both the original and optimized cutoff values, in combination with herd-level seropositivity status according to Bio K432_≥ 50_

When applying Bio K432_≥ 50,_ seroprevalence among adult cows ranged from 6.7% to 34.5% in BTM negative herds, from 69.7% to 89.2% in BTM positive herds and from 25.9% to 75.9% in BTM indeterminate herds, respectively. The distribution of the 14 herds according to the ID-screen ELISA with both original and optimized cutoff values on BTM, in combination with the seropositivity for Bio K432_≥ 50_ on these farms is represented in Fig. 1. When using the Bio K432_≥ 80_, seroprevalence ranged between 4.8% and 12% among adult cows in BTM negative herds, and between 20.8% and 62.2% in BTM positive herds, respectively. For the BTM indeterminate herds, seroprevalence varied between 6.9% and 69%. An overview of the S/P% in the different BTM categories for both lactating and dry cows with both the original and optimized cutoffs, as well as PCR results for all tested animal groups is provided in Table 3. In total 5/124 pooled NS samples were positive. All positive pooled NS samples were detected in two BTM positive herds. In herd 9, one positive pool was found in calves and one in youngstock. In herd 14, one positive pool was found in calves and two in youngstock. Additionally PCR results from one pooled NS sample (1/61) and one pooled VS sample (1/61) of the lactating animals were indiscriminate in the same two BTM positive herds.

**Table 3:** An overview of the percentage of seropositives in the different BTM categories for both lactating and dry cows with both the original and optimized cutoffs, as well as the positive PCR pools for all tested animal groups in 14 dairy herds (Belgium, 2024)

				Seropositives Bio K432_≥ 80_ (%)			Seropositives Bio K432_≥ 50_ (%)						
Herd	BTM S/P% ID Screen	N° youngstock sampled	N° calves sampled	Lact	Dry	Total adults*	Lact	Dry	Total adults	NS PCR pos**	NS PCR indis***	VS PCR pos	VS PCR indis
BTM negative (S/P% <30%)													
3	4.2	20	7	12.5 (3/24)	0 (0/5)	10.3 (3/29)	41.7 (10/24)	0 (0/5)	34.5 (10/29)	0	0	0	0
1	4.5	6	6	6.7 (1/15)	0 (0/6)	4.8 (1/21)	20 (3/15)	0 (0/6)	14.3 (3/21)	0	0	0	0
4	4.97	11	7	6.9 (2/29)	0 (0/1)	6.7 (2/30)	6.9 (2/29)	0 (0/1)	6.7 (2/30)	0	0	0	0
2	12.53	14	4	11.8 (2/17)	0 (0/5)	9.1 (2/22)	23.5 (4/17)	0 (0/5)	18.2 (4/22)	0	0	0	0
6	24.01	11	5	10 (2/20)	20 (1/5)	12 (3/25)	25 (5/20)	20 (1/5)	24 (6/25)	0	0	0	0
BTM indeterminate (S/P% 30–50%)													
7	46.1	8	2	12.5 (2/16)	ND	12.5 (2/16)	31.3 (5/16)	ND	31.3 (5/16)	0	0	0	0
5	48.97	16	7	6.7 (3/45)	7.7 (1/13)	6.9 (4/58)	20 (9/45)	46.2 (6/13)	25.9 (15/58)	0	0	0	0
13	48.98	17	6	65.4 (17/26)	100 (3/3)	69 (/29)	73.1 (19/26)	100 (3/3)	75.9 (22/29)	0	0	0	0
BTM positive (S/P% ≥50%)													
10	54.59	11	3	23.8 (5/21)	0 (0/3)	20.8 (5/24)	76.2 (16/21)	66.7 (2/3)	75 (18/24)	0	0	0	0
8	70.26	13	3	63.6 (21/33)	50 (2/4)	62.2 (23/37)	87.9 (29/33)	100 (4/4)	89.2 (33/37)	0	0	0	0
12	71.74	18	5	42.1 (16/38)	75 (3/4)	45.2 (19/42)	71.1 (27/38)	75 (3/4)	71.4 (30/42)	0	0	0	0
14	87.99	27	11	55.6 (20/36)	50 (2/4)	55 (22/40)	77.8 (28/36)	100 (4/4)	80 (32/40)	1	1	0	0
11	89.18	15	6	53.8 (14/26)	100 (3/3)	58.6 (17/29)	80.8 (21/26)	100 (3/3)	82.8 (24/29)	0	0	0	0
9	89.29	9	3	60 (18/30)	66.7 (2/3)	60.6 (20/33)	70 (21/30)	66.7 (2/3)	69.7 (23/33)	1	0	0	1

*Abbreviations*: *BTM* Bulk tank milk, *PCR* Polymerase chain reaction, *S/P%* Sample-to-positive percentage, *NS* Nasal swab, *VS* Vaginal swab, *ND* Not done, *N°* Number

*Total adults = sum of lactating and dry animals

**Positive PCR: Ct-values < 37

***Indiscriminate PCR: Ct-values: ≥37

## Discussion

The objective of the current study was to explore the potential of the ID-screen BTM antibody ELISA as a screening test for *M. bovis* in dairy herds not currently facing an epidemic period of respiratory disease or mastitis, by comparing BTM test results with PCR testing and serology of individual animals in different age categories. For the purpose of this study, farms were categorized by the ID-screen ELISA on BTM using a combination of the manufacturer’s guidelines and the optimized cutoff values described by [13], into one of three categories: BTM negative (S/P% <30%), BTM positive (S/P% ≥50%) and BTM indeterminate (S/P% 30–50%).

The primary finding of this study is that categorizing herds by BTM outcomes may be a useful screening approach. Based on the BTM categorization, three main observations can be made. First, the 30% S/P% cutoff used to categorize herds into the BTM negative group (with previously estimated 91.4% Se and 67.2% Sp) appears suitable for BTM screening purposes, as it minimizes the risk of false negatives [13]. Yet, although seropositivity was generally low in BTM negative herds, still up to 34% of the adults (herd 3) were seropositive according to Bio K432_≥ 50_. However, with an estimated 82.6% Se and 92.5% Sp of the used serum ELISA test, these seropositive results could be partly explained by the limitation of the test (false positive results) [18]. In addition, given that no antigen was detected in any age group on these BTM negative herds, the 30% S/P% could be used to classify herds as ‘Unsuspected of *M. bovis’*. Second, the optimized cutoff value (S/P% ≥50%) was used to make the BTM positive group. This cutoff results in a higher Sp (83.4% with a respective Se of 89.0%), which reduces the number of false positive herds [13]. In the BTM positive herds, we clearly observed seropositivity in all adult groups, including the dry cows. In addition, antigen was found in two of these BTM positive herds, whereas this was not retrieved in any of the other herds. Given both the high levels of BTM antibodies and high seroprevalence observed in BTM positive herds, these herds can be considered seropositive for *M. bovis*. As such, they could initially be classified as ‘Likely infected with *M. bovis’*. However, if solely based on ELISA results, it remains unclear whether these seropositive herds are experiencing an active infection. For these farms, it is also possible that they have experienced an infection in the past, but are no longer infectious, although asymptomatic or chronically infected animals may still be present [19]. To confirm an active infection, the pathogen should be cultivated through culture based methods or by detecting genetic material of *M. bovis* through PCR. However, the third and most intriguing finding regards to the four herds with BTM values around 50%. One herd (herd 10) was classified as a positive herd by using this BTM cutoff (S/P% ≥50) and the profile of that herd serology wise completely fitted the profile of the other BTM positive herds when using Bio K432_≥ 50_. In contrast, the three herds slightly below the BTM cutoff of 50% (BTM indiscriminate herds) displayed varying serological profiles according to Bio K432_≥ 50_. One herd (herd 13) had high seropositivity levels, whereas two other herds (herds 5 and 7) had seropositivity levels comparable to the BTM negative herds. As mentioned above, herd 13 was reclassified to BTM-indiscriminate between sampling occasions, which might indicate that this herd had experienced active circulation prior to this study and could explain the high seroprevalence. However, antigen was not found in any of the age categories within these intermediate herds. Yet, as seroprevalence can be high, these intermediate herds could initially be classified as ‘Suspected of *M. bovis’*. Both in suspected herds and herds which are likely infected with *M. bovis*, additional testing would be recommended to determine whether there is active circulation. In general, based on our findings, the 30% cutoff is the most appropriate threshold for broad screening as it reduces the chance of missing potentially exposed or previously infected herds. The 50% cutoff, due to its higher specificity, can be used to more reliably classify a farm as truly positive. By using a combination of both cutoffs, first identifying herds below 30% as clearly negative, above 50% as clearly positive, and herds in the intermediate group (30-50%) as suspected, misclassification can be minimized. 

Regarding this additional testing, it is noteworthy to mention that *M. bovis* antigen was only clearly detected in NS from either calves or youngstock. In lactating animals only two indiscriminate PCR results were observed in NS and VS. In theory indiscriminate results may be false positives or due to a lower number of bacteria in these adult animals, reflecting carriers or intermittent shedding, or it can be due to detection of non-viable bacterial DNA [20]. In the study of [21] they identified calves between 30 and 60 days of age as an interesting group to sample, which is in line with the results from our study. In addition, another study detected *M. bovis* in NS and deep nasopharyngeal swabs (DNS) from calves on farms where there had not been any case of clinical *M. bovis* mastitis in the cows in previous months [6]. These results, together with our findings, underscore the necessity of repeated calf monitoring as a key element in effectively screening and monitoring herd’s infection status. The efficacy of this screening may be increased when sampling is done in clinically affected animals. In our population, however, no calves/youngstock showed clinical signs, and selection was done at random. Given the large prevalence of subclinical pneumonia in *M. bovis* infected herds, selecting appropriate animals for additional sampling by means of thoracic ultrasound may be advisable [22]. As described by Feenstra, et al [23], vaginal swabs can be utilized to detect M. bovis in cattle, although the prevalence of detection may vary depending on the stage of infection and the presence of clinical signs. In the present population, vaginal swabs did not appear an added value, which may be due to the fact that we were not dealing with recently or clinically affected herds.

Given that this was a pilot study performed on a limited number of commercial dairy farms under field conditions, there are several limitations to be considered. First, the negative PCR results from NS and VS in adult cows could have been partly due to limitations of the used sampling technique. The tonsillar crypts are regarded as the primary site of *M. bovis* colonization and persistence in carriers colonization are the tonsils, but are difficult to sample in live animals [15]. The NS used in the present study may with a 15 cm length, have been too short to sample the pharyngeal tonsillar area, which may have reduced sensitivity [21, 24]. Another limitation was that, for budgetary reasons, serology in calves was not performed. Given that they are likely a main driver of infection within a herd, monitoring them as sentinel animals for antibodies may provide substantial added value. Also, antigen testing on VS in dry cows could not be done as some farmers objected due to a perceived risk of abortion. Still, given the high antibody titers found in the dry cows, this would be an interesting group for further testing. Further, not having included PCR testing on BTM may have been a limitation as a previous prevalence study in the same country showed that BTM antibody positive herds and BTM antigen positive herds were not associated [8]. In addition, we used a pooling technique for PCR analysis which may have reduced diagnostic accuracy. However, we estimate this to be limited as the used pooling technique was previously evidenced not to reduce diagnostic accuracy [20]. The present study was also subject to selection bias, related to herd size and the first come first serve basis of the enrolment. We opted to only include smaller herds in order to assure a sufficient percentage of the herd could be tested within budget. Still, the average herd size in the present study was close to the national average.

The authors would like to underscore that the present results apply to herds with no clinical presentation of *M. bovis* infection, hereby representing subclinically infected or not recently infected herds. Diagnostic accuracy of the explored tests may be much larger when applied in clinically affected herds, especially when targeted to ill animals. Taking the limitations of the present study into account, and based on the above introduced classification into (1) unsuspected, (2) suspected and (3) likely infected with *M. bovis*, it may be appropriate to install different sampling strategies for each of these tentative categories, hereby economizing resources. For the unsuspected group, no further sampling may be advised as this would only have a probability to incorrectly classify them as negative when using the manufacturer’s cutoff recommendation (cutoff ≥ 30%) [13]. For the suspected group, additional serological testing could be beneficial to help confirm the seropositive status, with our results indicating that testing dry cows could be particularly relevant. Testing of youngstock could also provide valuable insights as was also mentioned by [25]. Since the presence of antibodies has a 2–3 week lag period after acute infection and since it doesn’t ensure active circulation, active infection should be confirmed by antigen testing in both suspected herds and herds with a confirmed seropositive status. For sampling, a pooled sample of DNS taken from calves and youngstock may be recommended as a cost effective method. The role of BTM PCR testing needs further exploration, as it has shown to detect ongoing infection in lactating animals, but may miss the target group of cows with (sub)clinical mastitis [6]. Once antigen is detected, whether in BTM, during autopsy or through DNS testing, the herd can be definitively classified as “infected”.

## Conclusion

The findings of the present study suggest that based on BTM testing with the ID-screen ELISA, using the manufacturer’s (S/P% <30%) and optimized cutoff (S/P% ≥50%), herds can be classified in three groups, namely: unsuspected, suspected and likely infected with *M. bovis*. A possible testing strategy may be to first test by BTM antibody ELISA screening and take follow-up samples only in the suspected and likely infected herds. The preliminary results of the present study suggest that these should include antigen testing on DNS samples from calves/youngstock as well as serology from lactating and especially dry animals.

## Supplementary Information

Supplementary Material 1.

## Data Availability

Data is available from the corresponding author on reasonable request.

## Abbreviations

BTM: Bulk tank milk
ELISA: Enzyme-Linked Immunosorbent Assay
PCR: Polymerase chain reaction
M. bovis: *Mycoplasma bovis*
S/P%: Sample-to-positive percentage
VS: Vaginal swab
NS: Nasal swab
Ct: Cycle treshold
Med: Median
IQR: Interquartile range
SD: Standard Deviation
DNS: Deep nasopharyngeal swab
