# Supplementary material for: Bulk tank milk ELISA as screening test for Mycoplasma bovis: herd classification based on serology and PCR testing of different age groups
Source: Ir Vet J. 2026 Jan 17;79:12. doi: 10.1186/s13620-025-00327-x (PMC12874687; doi:10.1186/s13620-025-00327-x)
Supplement: Supplementary file 1 — Supplementary Material 1. [file 13620_2025_327_MOESM1_ESM.docx]

**Supplementary table 1**: Overview of BTM ID-Screen S/P% values for the initial 50 Belgian dairy herds, including results from a second BTM sampling around the time of the farm visit for the 14 selected herds.

| Herd | BTM S/P% ID-Screen  First sampling | Corresponding study herd number | BTM S/P% ID-Screen Second sampling |
| --- | --- | --- | --- |
| 1 | 3.46 |  |  |
| 2 | 4 |  |  |
| 3 | 4 |  |  |
| 4 | 4.1 | 1 | 4.5 |
| 5 | 4.1 |  |  |
| 6 | 5.38 | 3 | 4.21 |
| 7 | 5.38 |  |  |
| 8 | 6.02 |  |  |
| 9 | 6.66 | 4 | 4.97 |
| 10 | 8.04 |  |  |
| 11 | 8.36 |  |  |
| 12 | 8.47 |  |  |
| 13 | 9.43 |  |  |
| 14 | 9.54 |  |  |
| 15 | 9.86 |  |  |
| 16 | 15.29 | 2 | 12.53 |
| 17 | 15.61 |  |  |
| 18 | 15.93 |  |  |
| 19 | 24.77 | 6 | 24.01 |
| 20 | 35.82 |  |  |
| 21 | 43.74 | 5 | 48.97 |
| 22 | 45.44 |  |  |
| 23 | 45.66 | 7 | 46.1 |
| 24 | 60.58 | 8 | 70.26 |
| 25 | 61.32 |  |  |
| 26 | 61.64 |  |  |
| 27 | 63.56 |  |  |
| 28 | 73.47 |  |  |
| 29 | 73.68 |  |  |
| 30 | 77.09 |  |  |
| 31 | 78.48 |  |  |
| 32 | 78.48 |  |  |
| 33 | 79.97 |  |  |
| 34 | 80.29 |  |  |
| 35 | 81.03 | 11 | 89.18 |
| 36 | 81.03 |  |  |
| 37 | 82.31 |  |  |
| 38 | 82.63 |  |  |
| 39 | 83.48 |  |  |
| 40 | 85.4 | 10 | 54.59 |
| 41 | 88.39 | 12 | 71.74 |
| 42 | 91.26 |  |  |
| 43 | 97.34 | 9 | 89.29 |
| 44 | 99.71 | 14 | 87.99 |
| 45 | 101.28 |  |  |
| 46 | 104.26 |  |  |
| 47 | 105.33 |  |  |
| 48 | 115.24 |  |  |
| 49 | 128.02 | 13 | 48.98 |
| 50 | 143.37 |  |  |
